# Physiological rhythms and metabolic regulation: Shining light on skeletal muscle

**DOI:** 10.1113/EP091890

**Published:** 2025-01-29

**Authors:** James A. Betts, Kelly A. Bowden Davies, Harry A. Smith, John A. Hawley

**Affiliations:** ^1^ Centre for Nutrition, Exercise and Metabolism University of Bath Bath UK; ^2^ Department of Sport and Exercise Sciences, Institute of Sport Manchester Metropolitan University Manchester UK; ^3^ Exercise and Nutrition Research Program, Mary MacKillop Institute for Health Research Australian Catholic University Melbourne Victoria Australia

**Keywords:** chrono‐nutrition, circadian, fasting, infradian, meal timing, time‐restricted eating, ultradian

## Abstract

Metabolic regulation is essential for maintaining homeostasis in response to fluctuating dietary nutrient availability. In this review, we explore how metabolic health can be affected by the temporal alignment between daily behavioural patterns (e.g., eating, physical activity and sleep) and recurring cycles in underlying physiology (e.g., ‘circadian’ rhythms). Misalignment within and/or between these patterns and cycles can lead to metabolic dysregulation, increasing the risk of chronic disease states such as obesity, type 2 diabetes and cardiovascular disease. Conversely, metabolic health can be improved by strategically aligning certain behavioural patterns with endogenous rhythms in physiology. Dietary interventions based upon this reasoning are referred to as chrono‐nutrition strategies. Skeletal muscle is an important tissue in relation to both whole‐body metabolism and behaviour and plays a central role in how physiological rhythms respond to the timing of nutrient delivery/availability. Few studies have examined rhythms in metabolism within human skeletal muscle, providing opportunities to advance current understanding of how nutrient timing affects muscle metabolism.

## Abstract (highlights)

- **What is the topic of this review?**
  This review discusses the interactions between rhythms in human physiology and factors related to daily eating patterns to reveal how metabolic health can be targeted using chrono‐nutrition.
- **What advances does it highlight?**
  Metabolic regulation is inherently linked to physiological rhythms in metabolism and behaviour, with human skeletal muscle representing a major site for many of these responses. Scheduling the timing of daily meals according to underlying rhythms in physiology can impact skeletal muscle metabolism and impart metabolic health benefits.

## 1 INTRODUCTION

Metabolic regulation describes the mechanisms through which an intermittent delivery of dietary nutrients is channelled within and between tissues to meet the constantly fluctuating physiological requirements of an organism (Frayn, 2010). This variance in nutrient and energy flux over time poses a challenge to cellular and whole‐body homeostasis such that physiology and metabolism are heavily influenced by the alignment between daily light–dark, wake–sleep, activity–rest and fed–fasted cycles (Dibner & Schibler, 2018; Ekmekcioglu & Touitou, 2011). These cyclic states are anticipated by various endogenous rhythms in our physiology (described subsequently) that help to synchronize metabolism and behaviour to our environment throughout each day (Johnston, 2014; McGinnis & Young, 2016), with wakefulness, activity and feeding naturally scheduled to coincide during daylight hours in humans (Gerhart‐Hines & Lazar, 2015; Longo & Panda, 2016).

Our daily pattern of exposure to varying ambient light conditions is the primary factor that coordinates endogenous rhythms in physiology (Meng et al., 2023), and light can also directly exert acute effects on metabolism independent of those rhythms (Rao & Xue, 2024). Consequently, a regular schedule of early daytime light exposure is generally recommended (Brown et al., 2022), because mistimed light exposure later in the evening has been independently associated with cardiometabolic morbidity and mortality (Kim et al., 2022; Windred, Burns, Lane, et al., 2024; Windred, Burns, Rutter, et al., 2024). Beyond these influences of light per se, the entrainment of our timing system also depends on the alignment of light exposure relative to other environmental time cues, such as nutrition and physical activity. Asynchrony between our endogenous rhythms and these various environmental cues can therefore misalign this timing system, compromise metabolic control (Lund et al., 2001) and contribute to poor metabolic health, which can increase the risk of obesity, insulin resistance, type 2 diabetes, cardiovascular disease and some cancers (Harmsen et al., 2021; Johnston, 2014; Skene et al., 2018; Xiao et al., 2023).

Feeding or eating behaviour play key roles in the nutritional status of an organism, with human meal patterns dictated by complex interactions between inherent timing mechanisms, food availability, hunger/satiety and social conventions. There is a growing appreciation that numerous physiological processes are profoundly affected by the inter‐related factors that characterize the timing of nutrition, including the time of day at which food intake begins and ends, the frequency and regularity of eating occasions, and the scheduling of nutrient intake relative to other daily events, such as sleep and physical exercise/activity ‐ all of which are collectively described under the heading of ‘chrono‐nutrition’ (Hawley et al., 2020). In this regard, our internal body clocks operate as a crucial interface between nutrition (i.e., energy/nutrient availability) and whole‐body homeostasis. Here, we advance the hypothesis that metabolic health can be improved by manipulating modifiable lifestyle factors such that they better align with and/or more effectively synchronize the underlying rhythms in our innate physiology.

## 2 CIRCADIAN NOMENCLATURE

**FIGURE 1:**
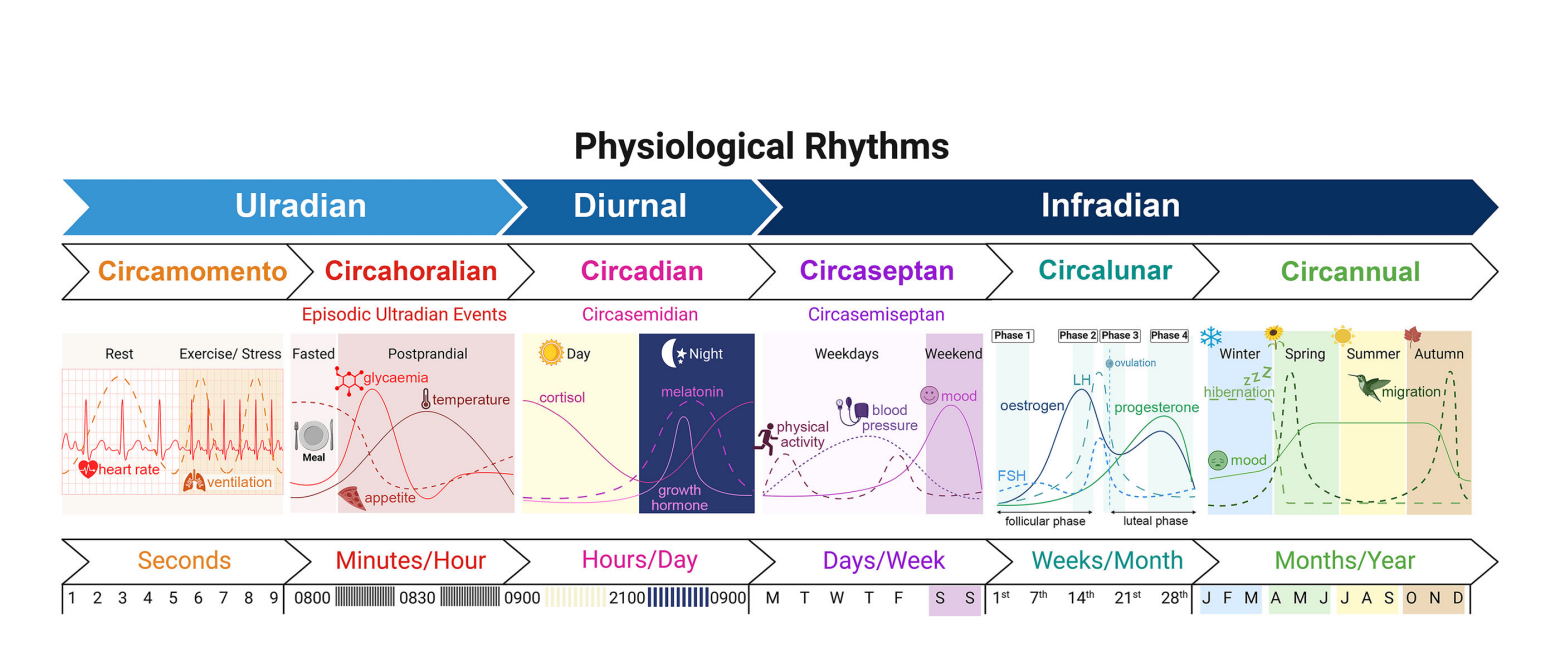
A summary of various rhythms in physiology, both under the broad headings that simply define frequencies of more than (ultradian), equal to (diurnal) or less than (infradian) one cycle per day and with the more traditional nomenclature used to specify endogenous rhythms with periods from seconds to decades. The examples of physiological rhythms shown include some that are more endogenously driven and others that are more acutely reactive to external cues, with most reflecting a combination of both and exhibiting rhythmicity over various time‐scales. Created in BioRender (2024) BioRender.com/s84n393.

Living organisms exhibit numerous physiological processes that follow repeating temporal patterns and are often loosely described as ‘circadian rhythms’. However, that particular term defines those cycles that are both endogenously synchronized (i.e., independent of external stimuli) and that recur approximately once each solar day (Mohawk et al., 2012). In contrast, diurnal rhythms describe the net ∼24‐h pattern of physiological responses observed when the effects of external stimuli are superimposed over underlying circadian rhythms (Duffy & Dijk, 2002). Other rhythms are neither strictly circadian nor diurnal because they do not have frequencies approximating a 24‐h cycle (Figure 1). For example, ultradian rhythms can occur multiple times within a day, with periods lasting from minutes to several hours (Goh et al., 2019), perhaps repeated only twice daily (e.g., circatidal clocks; Wilcockson & Zhang, 2008). Conversely, infradian rhythms span multiple days, with periods that can be approximately weekly/bi‐weekly (Mutak & Hlupić, 2017), monthly, seasonal, yearly or even less frequent (i.e., multiannual; Alerstam & Backman, 2018).

With such a lexicon of varied and overlapping terminology, it is understandable why these cyclic patterns are sometimes collectively referred to as ‘biological rhythms’, partly to avoid specifying their distinct origins or time scales. However, many organismic variables that exhibit rhythmicity are fundamentally more psychological, behavioural or social in nature, as opposed to purely biological constructs. The term ‘physiological rhythms’ might therefore more accurately capture the shared characteristics specific to these rhythmic parameters by focusing simply on the fact that they each might have conferred some evolutionary advantage (function) as mechanisms through which the entire living system is regulated.

The importance of these endogenously synchronized rhythms for physiological function is evident in the expression patterns of molecular machinery throughout various organs, tissues and cell types in mammals (Albrecht, 2017; Dierickx et al., 2018). For example, as much as 16% of the transcriptome exhibits rhythmic daily expression in any given tissue (Zhang et al., 2014). However, almost all of what is currently known about rhythms in metabolism within specific tissues is based on preclinical investigations using non‐human models. For example, targeted mutations of genes involved in regulating endogenous rhythms in various strains of mice yield animals with a number of metabolic disorders (Sahar & Sassone‐Corsi, 2012). Although rodent models have been invaluable in establishing links between physiological rhythms and the metabolism within individual tissues, that taxonomic order of mammals differ fundamentally from humans in their temporal patterns of both behaviour and metabolism.

Behaviourally, rodents are nocturnal animals but are generally active throughout each 24‐h light–dark cycle to forage and graze continuously (Ellacott et al., 2010). This contrasts starkly with the typical diurnal human pattern of clearly defined rest periods at night in a postabsorptive state (Ruge et al., 2009), with repeated bolus meals within a 12–14‐h eating window often restricted to daylight hours (Hawley et al., 2020). Interestingly, however, although diurnal humans and nocturnal rodents are somewhat opposites in their rhythmic expression of skeletal muscle clock genes between day and night, this apparent interspecies difference is eliminated if the accumulation pattern of gene transcripts is instead considered relative to the daily transition between phases of rest and activity (thus potentially pointing towards an important regulatory role of muscle contractile activity; Gutierrez‐Monreal et al., 2020).

Metabolically, there are important scaling issues owing to interspecies differences in body mass and the proportions of different organ systems, with rodents having a high surface area and metabolic rate relative to their mass, hence a more relentless need to deliver nutrients constantly via their disproportionately large splanchnic organs (Suarez et al., 2004). In addition, there are known interspecies differences in substrate handling and oxidation, with mice fuelling the energetic demands of exercise through a greater reliance on blood‐borne substrates (i.e., glucose), whereas humans rely to a greater extent on intramuscular fuels (i.e., muscle and liver glycogen, intramuscular triacylglycerols; Hawley et al., 2020). Indeed, rodents respond to limited nutrient availability not only with hyperactivity but also with a direct upregulation of skeletal muscle fatty acid oxidation, whereas humans respond to the same stimulus by limiting spontaneous energy expenditure and downregulating genes and proteins involved in glucose disposal within skeletal muscle (Hall & Hanford, 1954; Tsintzas et al., 2006). Clearly, observations made in vitro or in rodents do not always reflect the metabolic responses to varied nutrient availability in vivo in humans (Atkinson et al., 2014; Hawley et al., 2020).

## 3 SKELETAL MUSCLE: MISSION CONTROL FOR METABOLIC REGULATION

Skeletal muscle is a key site of metabolic regulation and is involved in the coordinated disposal, degradation and synthesis of nutrients on a daily basis (Frayn, 2010), being responsible for the majority of oxidative and non‐oxidative metabolism of our dietary macronutrients (DeFronzo et al., 1981; Ferrannini et al., 1985; Meyer et al., 2002; Ruge et al., 2009). Emerging evidence from studies in rodents has explored how the timing of skeletal muscle contractile activity can be aligned with other daily cycles in terms of dark–light, sleep–wake and fasting–feeding (Martin et al., 2023). These animal studies demonstrate that daily phases of both feeding and the timing of physical activity can serve as powerful ‘zeitgebers’ (time‐givers) or time‐setting cues to synchronize underlying rhythms in metabolism. To date, however, studies of human physiology have tended to focus on whether whole‐body outcomes are affected by altered meal patterns or physical activity. How various temporal patterns of nutrient delivery and/or flux (i.e., energy intake and expenditure) can align with or synchronize physiological rhythms in human skeletal muscle metabolism remains to be determined.

In recent years, several laboratories have sampled human skeletal muscle around the clock to generate the first data characterizing temporal rhythms in the human skeletal muscle transcriptome and lipidome, both in vitro (human primary myotubes; Hansen et al., 2016; Perrin et al., 2015) and in vivo (human skeletal muscle biopsies; Harmsen et al., 2024; Held et al., 2020; Loizides‐Mangold et al., 2017; Lundell et al., 2020; Perrin et al., 2018; van Moorsel et al., 2016; Wefers et al., 2020, 2018). For example, we recently developed a protocol using a semi‐constant (diurnal) routine, in which human volunteers rest whilst receiving nutrition continuously throughout waking hours (i.e., an absence of physical activity or acute meal responses), with serial blood and muscle sampling throughout a 24‐h period. Transcriptomic analysis of these samples revealed high‐amplitude rhythmicity in ∼1000 genes (rhythmic expression was detected at the pre‐mRNA and/or mRNA level for ∼40% of the 13,377 genes quantified), with two clear clusters exhibiting anti‐phasic distribution at 12‐h intervals. Notably, the two peaks in gene transcript accumulation included genes used for immune function and inflammation at 0400 h and pathways related to muscle glucose metabolism and protein turnover at 1600 h (e.g., PI3K–AKT–mTOR signalling for insulin‐stimulated glucose uptake and muscle regeneration/apoptosis; Perrin et al., 2018). In addition, lipidomic analyses identified diurnal rhythms in lipid metabolites that also peak at 0400 h, particularly major membrane‐lipid species, such as the sphingolipids that are involved in insulin signalling and insulin resistance (Loizides‐Mangold et al., 2017). These findings have since been replicated independently (Held et al., 2020) and extended to comprehensively demonstrate similar rhythmicity in mitochondrial respiration (Gemmink et al., 2023), the skeletal muscle metabolome (Harmsen et al., 2022) and the alignment of skeletal muscle gene expression relative to systemic metabolites and endocrine responses (Smith et al., 2025). Moreover, the rhythmicity of human skeletal muscle metabolites and genes involved in amino acid transport can be modified by short‐term restriction of the daily eating window (without perturbing core clock gene expression; Lundell et al., 2020), whereas regular physical exercise has recently been shown to modify the diurnal pattern of skeletal muscle clock gene expression (thus clearly demonstrating the capacity of contractile activity to entrain endogenous physiological rhythms; Harmsen et al., 2024).

Although these experimental models provide valuable ecologically valid information regarding diurnal patterns of metabolism over a standard pattern of day and night (including sleep), they do not all represent the kind of constant routine protocols necessary to characterize underlying physiological rhythms (Duffy & Dijk, 2002). Important questions therefore remain, such as whether nocturnal responses are dependent on the withdrawal of nutrition during the dark/sleep phase and therefore the extent to which the observed 24‐h rhythmicity is endogenously or exogenously driven.

## 4 CHRONO‐NUTRITION: IMPACT ON METABOLIC HEALTH

Over the past decade, there has been an increasing appreciation that the duration over which food is consumed each day can have marked effects on a variety of physiological processes. Such ‘timed eating’ or chrono‐nutrition strategies might therefore hold the potential to alter metabolic health favourably by scheduling nutrient intake according to the acrophases of endogenous rhythms in metabolism. Although there are numerous approaches to alter meal timing and therefore to manipulate nutrient availability and metabolic flux within skeletal muscle (along with other insulin‐sensitive tissues/organs), mounting evidence suggests that scheduled fasting to restrict the duration spent in the fed‐state each day might improve symptoms associated with metabolic disorders.

Traditional dietary approaches have tended to focus on chronic or continuous energy restriction, either by reducing the total amount of food consumed at all eating occasions and/or altering the types of foods in the diet (e.g., lower energy density), all without any need to adjust daily meal patterns/timing. In contrast, intermittent fasting is a popular dietary strategy whereby eating patterns are scheduled to accommodate specified and sometimes extended periods in the postabsorptive (fasted) state (often therefore indirectly reducing total energy intake). Time‐restricted eating (TRE) is a specific subcategory of intermittent fasting whereby food intake is limited to a defined eating window (i.e., the time between the first and last energy intake each day), generally reducing that period from the typical 12–14 h that span most of the waking day to 8–10 h (possibly without altering total energy intake). It should be emphasized that neither chronic/continuous energy restriction nor intermittent fasting is necessarily a chrono‐nutritive therapy per se, in that they need not regularly prescribe nutrients at set times of day in order to align with or synchronize endogenous rhythms in physiology. Instead, their therapeutic value and positive health outcomes might be mainly derived simply from chronic or intermittent energy restriction (Hawley et al., 2020).

For people with pre‐existing metabolic conditions (e.g., type 2 diabetes) or at risk of developing metabolic conditions, a growing body of evidence suggests that TRE can improve glucose and lipid metabolism and blood pressure and can contribute to improved overall health, including reduced risk of cardiovascular disease (Hawley et al., 2020; Smith & Betts, 2022). Early studies following TRE protocols in humans reported a 10–20% reduction in voluntary energy intake, thus it was not known whether the health benefits induced by this strategy were attributable to energy deficit (weight‐loss) and/or other mechanisms. However, a proof‐of‐concept study recently demonstrated that, in men with prediabetes, 5 weeks of early TRE (i.e., eating window of only 6 h·day^−1^, from 0800 h to 1400 h) increased insulin sensitivity and β‐cell responsiveness, whilst lowering blood pressure and oxidative stress, independent of weight‐loss (Sutton et al., 2018). However, such an eating regimen is extreme and unlikely to be adopted or adhered to by many ‘at risk’ populations. Until recently, few studies had examined the effects of TRE in humans under free‐living conditions, neither had any research explored isoenergetic ‘early’ versus ‘late’ TRE protocols to unravel the mechanistic underpinning of how these eating patterns might alter flux through tissues such as skeletal muscle. Indeed, restricting nutrition intake (especially protein) within a relatively brief (∼8 h·day^−1^) eating window might compromise the net daily capacity for protein synthesis, which could predispose to sarcopenia, loss of physical function and impaired metabolic health in the long term. However, the results of two recent studies examining isoenergetic and iso‐nitrogenous TRE (8 vs. 12 h·day^−1^ eating windows) indicate that, in the short term (i.e., 10 days), such dietary protocols do not impair rates of muscle protein synthesis in overweight/obese men (Kouw et al., 2024; Parr et al., 2023), whereas another free‐living study reports that reducing the daily eating window from ≥14 to <10 h for 3 weeks can improve 24‐h glucose homeostasis amongst men and women with type 2 diabetes (Andriessen et al., 2022).

## 5 CONCLUSIONS AND FUTURE DIRECTIONS

Despite recent progress in the field of circadian biology, substantial gaps remain in the current understanding of how nutrient timing and physical activity might interact to affect muscle metabolism and overall cardiometabolic health. Given established links between the timing of modifiable lifestyle factors and numerous chronic diseases, it will be valuable to extend knowledge in several key areas.

One logical step to provide further insight would involve using enteral delivery of nutrients throughout a 24‐h day (including during sleep) to examine whether the apparent nocturnal responses described earlier (Loizides‐Mangold et al., 2017; Perrin et al., 2018; Smith et al., 2025; Templeman et al., 2021) are dependent on the withdrawal of nutrition at night. The continuous delivery of nutrition represents the maximum possible frequency of feeding over 24 h without eliciting the acute post‐prandial responses typical of daily eating patterns. In contrast to continuous nutrient delivery, a model of daytime bolus feeding (more typical of habitual meal patterns for most people) would be another approach to further our understanding of how nutrient timing impacts physiological rhythms. Continuous enteral feeding provides a model for the investigation of other zeitgebers (i.e., whilst acute meal responses are absent). Although protocols to date have used fixed sedentary conditions to enable the characterization of rhythmic patterns in basal/resting conditions, complete inactivity does not represent the context in which the human genome was shaped (i.e., obligatory physical activity required for survival). It will therefore be important for further research to investigate how muscle contractile activity per se (in addition to its temporal distribution across the day and alignment with feeding patterns) relates to 24 h rhythms in human skeletal muscle metabolism. Certainly, recent studies have now clearly demonstrated the potential for muscle contraction to alter the rhythmic expression of genes directly in skeletal muscle, both after an acute bout of exercise (Small et al., 2020) and following regular exercise training (Harmsen et al., 2024), hence it seems a logical hypothesis that rhythms in physiological functions/outcomes (e.g., metabolic fluxes) might also then be responsive to the precise timing of daily exercise.

Despite growing evidence that TRE can improve metabolic health, our understanding of its impact on skeletal muscle and other insulin‐sensitive tissues remains limited, particularly in relation to muscle mass, protein synthesis and physical function. In this regard, it will be important to study these outcomes in populations such as the elderly or infirm, who might stand to benefit in some ways from well‐aligned feeding patterns but might also exhibit a degree of anabolic resistance that is not well‐suited to extended periods of fasting. In addition, there is a lack of research examining the nutrient‐specific effects of TRE. In the fullness of time, chrono‐nutrition strategies might be most effective when integrated alongside more conventional strategies focused on the amount and type of foods consumed. For example, just as TRE can be effective for weight‐loss and health‐gain but can be difficult to maintain in the long term, the same might also be said for other popular strategies, such as ketogenic diets. Perhaps a balanced combination of the two may confer the benefits of both approaches without having to fast completely, such as by only restricting carbohydrate at certain times (i.e. time‐restricted carbohydrate) or by allowing carbohydrate intake only coincident with physical/contractile activity. Certainly, the intake of a number of nutrients typically exhibit an uneven distribution across each day [e.g., breakfast tends to be rich in carbohydrates but relatively low in protein (USDA Agricultural Research Service, 2012), whereas alcohol tends to be consumed later in the day], hence the most effective and practical scheduling of TRE should take into account different rhythms in metabolism according to the real‐time supply and demand specific to each individual nutrient.

In conclusion, in this review, we have highlighted the intricate and interactive relationships between physiological rhythms and metabolic regulation, with a particular focus on skeletal muscle. We highlight the potential of chrono‐nutrition and the timing of nutrient intake to align with physiological rhythms, suggesting that the temporal distribution of meals (e.g., TRE) and physical activity can enhance metabolic health outcomes. Future research on continuous nutrient delivery and chrono‐nutrition interventions holds promise for advancing our understanding in this area and improving metabolic health.

## AUTHOR CONTRIBUTIONS

Kelly A. Bowden Davies was responsible for the concept of the review. All authors contributed to the design and writing of the manuscript. All authors approved the final version of the manuscript and agree to be accountable for all aspects of the work in ensuring that questions related to the accuracy or integrity of any part of the work are appropriately investigated and resolved. All persons designated as authors qualify for authorship, and all those who qualify for authorship are listed.

## CONFLICT OF INTEREST

J.A.B. is an investigator receiving research grants funded by biotechnology and biological sciences research council (BBSRC), medical research council (MRC), national institutes of health and care research (NIHR), British Heart Foundation, Rare Disease Foundation, EU Hydration Institute, GlaxoSmithKline, Nestlé, Lucozade Ribena Suntory, ARLA foods, Cosun Nutrition Center, American Academy of Sleep Medicine Foundation, Salus Optima (L3M Technologies Ltd) and the Restricted Growth Association; has completed paid consultancy for PepsiCo, Kellogg's, SVGC and Salus Optima (L3M Technologies Ltd); is Company Director of Metabolic Solutions Ltd; receives an annual honorarium as a member of the academic advisory board for the International Olympic Committee Diploma in Sports Nutrition; and receives an annual stipend as Editor‐in‐Chief of *International Journal of Sport Nutrition & Exercise Metabolism*. H.A.S. has received funding from the Sleep Research Society Foundation and The Rank Prize Funds and is a former employee of ZOE Ltd, from which he received share options as part of this employment and for which he still holds an unpaid consultancy role. K.B.D. is an investigator on research grants funded by BBSRC, MRC and Abbott Laboratories.
